# Anatomical variations in the insular cortex in individuals at a clinical high-risk state for psychosis and patients with schizophrenia

**DOI:** 10.3389/fpsyt.2023.1192854

**Published:** 2023-07-05

**Authors:** Tsutomu Takahashi, Daiki Sasabayashi, Yoichiro Takayanagi, Yuko Higuchi, Yuko Mizukami, Yukiko Akasaki, Shimako Nishiyama, Atsushi Furuichi, Haruko Kobayashi, Yusuke Yuasa, Noa Tsujii, Kyo Noguchi, Michio Suzuki

**Affiliations:** ^1^Department of Neuropsychiatry, University of Toyama Graduate School of Medicine and Pharmaceutical Sciences, Toyama, Japan; ^2^Research Center for Idling Brain Science, University of Toyama, Toyama, Japan; ^3^Arisawabashi Hospital, Toyama, Japan; ^4^Health Administration Center, Faculty of Education and Research Promotion, Academic Assembly, University of Toyama, Toyama, Japan; ^5^Department of Child Mental Health and Development, Toyama University Hospital, Toyama, Japan; ^6^Department of Radiology, University of Toyama Graduate School of Medicine and Pharmaceutical Sciences, Toyama, Japan

**Keywords:** magnetic resonance imaging, at-risk mental state, early psychosis, cognition, insula, gyrification

## Abstract

**Introduction:**

Since the number of insular gyri is higher in schizophrenia patients, it has potential as a marker of early neurodevelopmental deviations. However, it currently remains unknown whether the features of the insular gross anatomy are similar between schizophrenia patients and individuals at risk of psychosis. Furthermore, the relationship between anatomical variations in the insular cortex and cognitive function has not yet been clarified.

**Methods:**

The gross anatomical features (i.e., the number of gyri and development pattern of each gyrus) of the insular cortex were examined using magnetic resonance imaging, and their relationships with clinical characteristics were investigated in 57 subjects with an at-risk mental state (ARMS) and 63 schizophrenia patients in comparison with 61 healthy controls.

**Results:**

The number of insular gyri bilaterally in the anterior subdivision was higher in the ARMS and schizophrenia groups than in the control group. The schizophrenia group was also characterized by a higher number of insular gyri in the left posterior subdivision. A well-developed right middle short insular gyrus was associated with symptom severity in first-episode schizophrenia patients, whereas chronic schizophrenia patients with a well-developed left accessory gyrus were characterized by less severe cognitive impairments in motor and executive functions. The features of the insular gross anatomy were not associated with clinical characteristics in the ARMS group.

**Discussion:**

The features of the insular gross anatomy that were shared in the ARMS and schizophrenia groups may reflect a vulnerability to psychosis that may be attributed to anomalies in the early stages of neurodevelopment. However, the contribution of the insular gross anatomy to the clinical characteristics of schizophrenia may differ according to illness stages.

## Introduction

The insular cortex, a ‘limbic integration cortex’ (1) located at the base of the sylvian fissure, is characterized by anatomical and functional complexities with large individual differences in the gross gyral pattern (2, 3). Previous studies reported an underdeveloped or absent accessory gyrus (AG) and middle short gyrus (MSG) in the anterior subdivision in 50–70% of human brains and an absent posterior long gyrus (PLG) in the posterior subdivision in approximately 10–20% (4–6). Although it currently remains unknown whether these variations are of functional significance, the findings of our recent magnetic resonance imaging (MRI) study on schizophrenia (7) revealed a larger number of insular gyri (particularly the AG, MSG, and PLG) and a relationship with positive symptomatology in first-episode patients. The insular cortex has been suggested to exhibit progressive reductions in gray matter during the early stages of psychosis (8, 9), whereas insular gyral patterns in schizophrenia were reported to be independent of illness stages (e.g., first-episode vs. chronic stage) (7). Therefore, the features of the gross insular anatomy in schizophrenia patients may reflect a stable trait associated with abnormal neurodevelopment during fetal insular gyration, which occurs during mid to late gestation (10, 11). However, it has not yet been established whether the features of the insular gross anatomy are similar between schizophrenia patients and individuals at risk of psychosis, and, thus, their potential as a marker of vulnerability to psychosis remains unknown.

Previous MRI studies on patients with a clinical high-risk state for psychosis [i.e., at-risk mental state (ARMS)] (12, 13), approximately 30% of whom develop psychosis within 2 years (14), have generally demonstrated similar gross brain morphological features related to early neurodevelopmental anomalies, such as widespread cortical hyper-gyrification (15) and an altered gross sulco-gyral pattern in the temporal (16) and orbitofrontal (17) regions, to those observed in schizophrenia (18, 19). Since these brain morphological features are also present in ARMS individuals without the later onset of psychosis (15–17), they indicate a general vulnerability to psychopathology. These biological traits have been suggested to play a role in the development of cognitive impairments (e.g., executive dysfunction) in ARMS individuals (18) and schizophrenia patients (20), supporting the presence of cognitive impairments prior to the onset of psychosis as a trait vulnerability marker (21, 22). However, to the best of our knowledge, the features of the insular gross anatomy in ARMS have not yet been examined using MRI, and it remains also unclear whether they are associated with cognitive function in ARMS or schizophrenia.

Therefore, the present MRI study examined variations in the gross insular anatomy (i.e., the number of gyri and development pattern of each gyrus) in schizophrenia, ARMS, and control groups. Based on previous findings obtained from an independent cohort of schizophrenia (7) as well as the gross brain morphological features shared between ARMS and schizophrenia (15–17), the number of insular gyri was predicted to be higher in both clinical groups than in the control group. We also investigated the relationships between the insular gross anatomy and clinical characteristics (e.g., cognitive function and symptom severity) in ARMS and the different illness stages of schizophrenia (i.e., first-episode and chronic stage).

## Materials and methods

### Participants

As summarized in Table 1, 63 schizophrenia patients, 57 individuals with ARMS, and 61 healthy controls were enrolled in the present study. Recruitment strategies (e.g., inclusion/exclusion criteria) and sample characteristics were fully described elsewhere (16, 23); none of the participants had a history of serious medical issues (e.g., thyroid disease, diabetes, and serious head injury), severe obstetric complications, or substance abuse. A clinical diagnosis of schizophrenia was confirmed at Toyama University Hospital by experienced psychiatrists using the Structured Clinical Interview for DSM-IV Axis I Disorders Patient Edition (24) and that of ARMS using the Comprehensive Assessment of ARMS (13). To examine the role of the illness stages, the schizophrenia group was further divided into two subgroups: first-episode [duration ≤1 year (*N* = 17)] and chronic [duration ≥3 years (*N* = 38)] (25, 26). Five ARMS subjects developed schizophrenia during the clinical follow-up (mean = 1.5 years after MRI scanning, SD = 2.6). At the time of MRI scanning, 14 of the 57 ARMS subjects (24.64%) were receiving a low dosage of antipsychotics for their relatively severe symptoms (e.g., rapid deterioration and suicidal risk) according to the International Clinical Practice Guidelines for Early Psychosis (27). Healthy controls with no personal or family history of neuropsychiatric disorders were recruited from the community or hospital staff and screened by the SCID-I Non-patient Edition (24).

**Table 1:** Demographic and clinical data of study participants.

	Controls (*N* = 61)	ARMS (*N* = 57)	Sz (*N* = 63)	Group difference
Male [%]	32 [52.5]	34 [59.6]	29 [46.0]	Chi-squared = 2.23, *p* = 0.329
Age (years)	25.6 ± 3.2	18.6 ± 4.3	28.0 ± 9.4	*F*(2, 178) = 34.93, *p* < 0.001; ARMS < Controls, Sz
Height (cm)	166.0 ± 8.3	164.4 ± 9.0	163.2 ± 8.4	*F*(2, 178) = 1.68, *p* = 0.190
Hand dominance (right/mixed/left)	40/15/6	35/17/5	52/9/2	Chi-squared = 7.73, *p* = 0.102
SES	6.2 ± 0.9	3.2 ± 1.4	4.2 ± 1.4	*F*(2, 178) = 92.20, *p* < 0.001; ARMS < Sz < Controls
Parental SES^a^	5.9 ± 0.9	5.0 ± 0.9	4.8 ± 1.4	*F*(2, 177) = 16.94, *p* < 0.001; ARMS, Sz < Controls
JART estimated IQ	110.2 ± 5.9	98.5 ± 9.7	99.5 ± 9.7	*F*(2, 178) = 34.35, *p* < 0.001; ARMS, Sz < Controls
Onset age (years)	–	–	22.4 ± 7.4	–
Illness duration (years)	–	–	5.5 ± 6.0	–
Antipsychotic medication				
Dose (Haloperidol equiv., mg/day)	–	2.5 ± 1.8 (*N* = 14)	11.3 ± 7.8 (*N* = 51)	*F*(1, 63) = 17.32, *p* < 0.001; ARMS < Sz
Type (typical/atypical/mixed)	–	1/12/1	1/45/5	Fisher’s exact test, *p* = 0.585
Duration (years)	–	0.7 ± 1.2 (*N* = 17)	5.2 ± 6.2 (*N* = 53)	*F* (1, 68) = 8.78, *p* = 0.004; ARMS < Sz
PANSS				
Positive	–	11.6 ± 3.2	13.9 ± 5.6	*F*(1, 118) = 7.45, *p* = 0.007; ARMS < Sz
Negative	–	15.3 ± 6.6	16.3 ± 5.6	*F*(1, 118) = 0.63, *p* = 0.428
General	–	30.2 ± 7.9	31.0 ± 9.7	*F*(1, 118) = 0.25, *p* = 0.619
BACS subdomain z-scores				Group-by-domain interaction, *F* (5, 590) = 6.29, *p* < 0.001
Verbal memory	–	−0.7 ± 1.6	−1.4 ± 1.4	*p* = 0.347
Working memory	–	−0.7 ± 1.3	−1.0 ± 1.4	*p* = 1.000
Motor function	–	−0.9 ± 1.4	−1.9 ± 1.5	*p* = 0.004; Sz < ARMS
Verbal fluency	–	−0.9 ± 1.5	−0.8 ± 1.1	*p* = 1.000
Attention and processing speed	–	−0.2 ± 1.4	−1.4 ± 1.5	*p* < 0.001; Sz < ARMS
Executive function	–	−0.3 ± 1.2	−0.8 ± 1.6	*p* = 0.840
SOFAS^a^	–	51.7 ± 10.2	48.2 ± 13.9	*F* (1, 117) = 2.55, *p* = 0.113
SCoRS global rating score^a^	–	5.3 ± 2.3	5.2 ± 2.5	*F* (1, 117) = 0.02, *p* = 0.899

Values represent means ± SDs unless otherwise stated. ARMS, at-risk mental state; BACS, Brief Assessment of Cognition in Schizophrenia; IQ, intelligence quotient; JART, Japanese version of National Adult Reading Test; PANSS, Positive and Negative Syndrome Scale; SCoRS, Schizophrenia Cognition Rating Scale; SES, socioeconomic status; SOFAS, Social and Occupational Functioning Assessment Scale; Sz, schizophrenia. ^a^Data missing for one schizophrenia patient.

The Committee of Medical Ethics of the University of Toyama (ID: I2013006) approved the present study. After obtaining a full description of the protocol of this study, all participants provided their written informed consent according to the guidelines of the Declaration of Helsinki. If a participant was younger than 20 years old, written consent was also obtained from a parent/guardian. None of the study participants overlapped with those in our previous gross insular study using 1.5-tesla MRI data (7).

### Clinical assessment

Clinical symptoms in the schizophrenia and ARMS groups were assessed at the time of MRI scanning by experienced psychiatrists using the Positive and Negative Syndrome Scale (PANSS) (28). As previously reported (29, 30), subjects were also administered the Brief Assessment of Cognition in Schizophrenia (BACS) (31), the Schizophrenia Cognition Rating Scale (SCoRS) (32), and the Social and Occupational Functioning Assessment Scale (SOFAS) (33) to assess socio-cognitive functions.

### MR imaging procedures

As detailed previously (16, 23), all subjects were scanned by a 3.0-tesla MR scanner (Magnetom Verio, Siemens, Erlangen, Germany) at Toyama University Hospital; a 3-D MPRAGE sequence was applied to obtain T1-weighted 1.2-mm consecutive sagittal images with a voxel size of 1.0 × 1.0 × 1.2 mm. The following parameters were used: TR/TE = 2300/2.9 ms, flip angle = 9°, field of view = 256 mm, and matrix = 256 × 256 pixels. Dr. View software (Infocom, Tokyo, Japan) was used to reconstruct the images obtained into 1-mm-thick coronal images perpendicular to the inter-commissural line after tilt correction.

### Assessment of the insular gross anatomy

**Figure 1:**
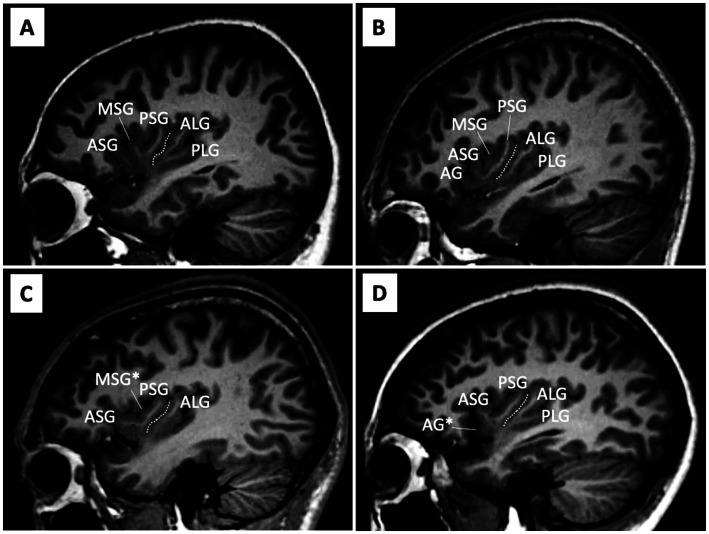
Anatomical variations in the insular cortex on sample MR images in the sagittal view. **(A-D)** Assessments of gyral development were mainly performed using consecutive sagittal slices, with references to simultaneously displayed coronal and axial views. The central insular sulcus (white dotted lines) subdivides the anterior (short insula) and posterior (long insula) subdivisions. The AG and MSG had large inter-individual variations in the degree of development [i.e., absent, underdeveloped (marked with an asterisk), or well-developed], while the ASG, PSG, and ALG were well-developed in all hemispheres. The PLG was generally present, but was absent in some hemispheres (e.g., hemisphere C). AG, accessory gyrus; ALG, anterior long gyrus; ASG, anterior short gyrus; MSG, middle short gyrus, PLS, posterior long gyrus; PSG, posterior short gyrus.

As previously reported (7), AG and MSG were classified as fully developed, underdeveloped (i.e., present, but not reaching the convex surface of the insular cortex), or absent (Figure 1 and Table 2). Since hypoplasia of the PLG is rarely observed in human brains (4, 6), it was classified as present or absent. Other major insular gyri [i.e., the anterior short gyrus (ASG), posterior short gyrus (PSG), and anterior long gyrus (ALG)] were well-developed in all hemispheres in subjects. Regarding the number of anterior and posterior insular gyri, only well-developed gyri were included in the count.

**Table 2:** Classification of insular gyri in the present study.

Insular gyri	Classification	Definitions and notes
Short insular gyri		
Accessory gyrus (AG)	Absent, underdeveloped, or developed	A gyrus branching from the anterior surface of ASG more than one-third below its upper end. When identifiable, the AG reaching and not reaching the convex surface of the insula were defined as developed and underdeveloped, respectively.
Anterior short gyrus (ASG)	Developed	Well-developed in all 362 hemispheres in the present sample.
Middle short gyrus (MSG)	Absent, underdeveloped, or developed	When identifiable, the MSG was classified with the same definition as the AG development classification.
Posterior short gyrus (PSG)	Developed	Well-developed in all 362 hemispheres in the present sample.
Long insular gyri		
Anterior long gyrus (ALG)	Developed	Well-developed in all 362 hemispheres in the present sample.
Posterior long gyrus (PLG)	Absent or present	No underdeveloped cases in the present sample of 362 hemispheres.

The classification was based on the criteria by Wysiadecki et al. (6).

These assessments were conducted by one rater (TT) who was blinded to the identities of the subjects. Intra- (TT) and inter-rater (TT and DS) reliabilities were both >0.81 for the developmental pattern (Cronbach’s *α*) and gyral number (intraclass correlation coefficients) in randomly selected 10 brains (20 hemispheres).

### Statistical analysis

The χ^2^ test or an analysis of variance (ANOVA) was used to compare demographic and clinical data between groups (Table 1).

The χ^2^ test or Fisher’s exact test was used to investigate differences in the gyral development of the AG, MSG, and PLG between groups. Comparisons of the total number of short (the AG, ASG, MSG, and PSG) and long (the ALG and PLG) insular gyri, which was log-transformed due to a non-normal distribution (Kolmogorov–Smirnov tests), were performed between groups using ANOVA with group and sex as between-subject variables and hemisphere as a within-subject factor. Post-hoc Scheffé’s tests were employed.

Spearman’s correlation analysis with the Bonferroni correction was performed to establish whether the number of short gyri was associated with demographic/clinical variables [intelligence quotient (IQ), onset age, illness duration, medication dose/duration, PANSS subscale scores, and SOFAS, SCoRS, and BACS scores]. The long insular cortex had two gyri in most hemispheres [*N* = 327/362 (90.3%)] and was not eligible for the correlation analysis. The effects of the development of insular gyri on these clinical variables were examined using the non-parametric Mann–Whitney *U* test (onset age and illness duration for schizophrenia; medication, SOFAS, SCoRS, and BACS executive function scores for both schizophrenia and ARMS; and BACS verbal/working memory scores for ARMS) or ANOVA (PANSS subscale scores and other BACS subdomain scores) based on the distribution of data (tested by Kolmogorov–Smirnov tests), where the development pattern (well-developed vs. underdeveloped or absent) was used as a between-subject factor. It was not possible to reliably assess the potential effects of the development of the PLG on clinical variables due to small number of subjects with an absent PLG (*N* ≤ 5 each in the schizophrenia and ARMS groups). Significance was defined as *p* < 0.05.

## Results

### Sample characteristics

No significant differences were observed in the sex ratio, height, or handedness between groups; however, subjects in the ARMS group were younger than those in the other groups (Table 1). The ARMS and schizophrenia groups had a lower IQ and socioeconomic status than the control group. Subjects in the schizophrenia group, who had more severe positive symptoms and cognitive impairments, received more medication than those in the ARMS group (Table 1).

### Insular gross anatomy of participants

**Figure 2:**
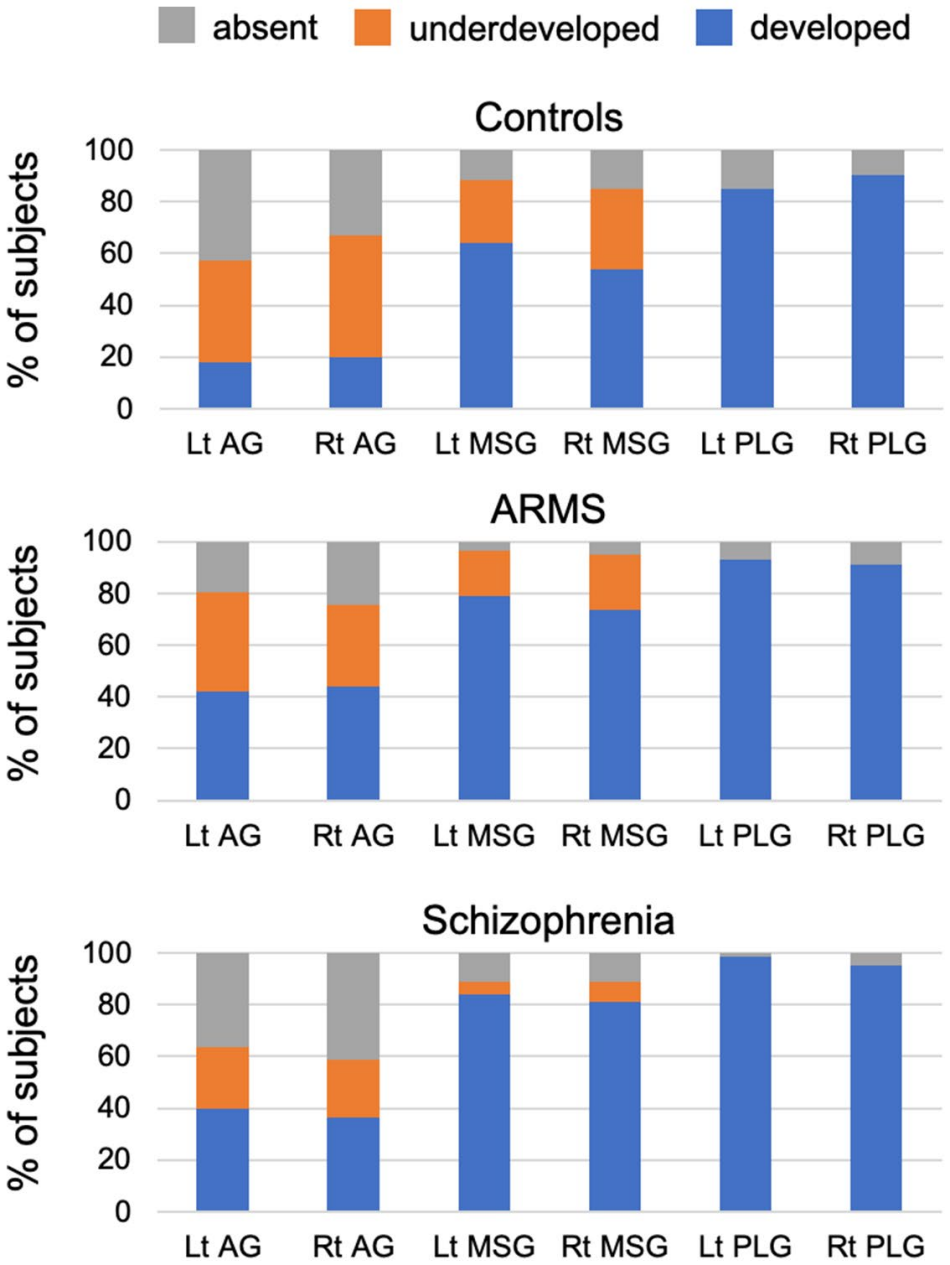
Degree of development of the accessory gyrus (AG), middle short gyrus (MSG), and posterior long gyrus (PLG) in control, at-risk mental state (ARMS), and schizophrenia groups in the present study.

The gyral development of the AG, MSG, and PLG significantly differed between the groups (Table 3 and Figure 2). The prevalence of well-developed gyri for the bilateral AG (left, χ^2^ = 7.05, *p* = 0.008; right, χ^2^ = 4.34, *p* = 0.037), bilateral MSG (left, χ^2^ = 6.60, *p* = 0.010; right, χ^2^ = 10.23, *p* = 0.001), and left PLG (Fisher’s exact test, *p* = 0.008) was higher in the schizophrenia group than in the control group. The bilateral AG (left, χ^2^ = 8.18, *p* = 0.004; right, χ^2^ = 8.01, *p* = 0.005) and right MSG (χ^2^ = 4.88, *p* = 0.027) were significantly more developed in the ARMS group than in the control group. Only the development of the left MSG differed between the schizophrenia and ARMS groups (Fisher’s exact test, *p* = 0.031), where the prevalence of an underdeveloped gyrus was higher in the ARMS group.

**Table 3:** Gross insular morphology of study participants.

	Controls (*N* = 61)	ARMS (*N* = 57)	Sz (*N* = 63)	Group differences
Number of short gyri				*F*(2, 175) = 14.81, *p* < 0.001; Controls < ARMS, Sz
Left	2.87 ± 0.64	3.23 ± 0.73	3.27 ± 0.63	
Right	2.79 ± 0.58	3.30 ± 0.78	3.19 ± 0.62	
Number of long gyri				*F*(2, 175) = 3.48, *p* = 0.033; Controls < Sz
Left	1.85 ± 0.36	1.96 ± 0.33	2.02 ± 0.22	
Right	1.92 ± 0.33	1.93 ± 0.32	1.97 ± 0.25	
AG (absent/underdeveloped/developed)				
Left	26/24/11	11/22/24	23/15/25	Chi-squared = 14.37, *p* = 0.006
Right	20/29/12	14/18/25	26/14/23	Chi-squared = 14.06, *p* = 0.007
MSG (absent/underdeveloped/developed)				
Left	7/15/39	2/10/45	7/3/53	Chi-squared = 12.578, *p* = 0.014
Right	9/19/33	3/12/42	7/5/51	Chi-squared = 14.42, *p* = 0.006
PLG (absent/present)				
Left	9/52	4/53	1/62	Fisher’s exact test, *p* = 0.020
Right	6/55	5/52	3/60	Fisher’s exact test, *p* = 0.573

Values represent means ± SDs unless otherwise stated. AG, accessory gyrus; ARMS, at-risk mental state; MSG, middle short gyrus; PLG, posterior long gyrus; Sz, schizophrenia.

The schizophrenia and ARMS groups had significantly more short gyri (Scheffé’s test, *p* < 0.001) than the control group (Table 3). Furthermore, the schizophrenia group had significantly more long gyri than the control group (Scheffé’s test, *p* = 0.033). The number of insular gyri did not significantly differ between the schizophrenia and ARMS groups. These results remained unchanged when age and medication (dose and duration) were used as covariates.

Neither the number of short [*F*(1, 53) = 0.48, *p* = 0.492] and long [*F*(1, 53) = 0.14, *p* = 0.707] gyri nor gyral development (χ^2^ ≤ 0.80, *p* ≥ 0.521) significantly differed between the first-episode (*N* = 17) and chronic (*N* = 38) schizophrenia subgroups. The results of the study remained essentially the same even when we excluded the ARMS subjects who were taking antipsychotics at the time of scanning.

### Relationships between features of the insular gross anatomy and clinical characteristics

PANSS positive [*F*(1, 15) = 8.17, *p* = 0.012] and general [*F*(1, 15) = 4.79, *p* = 0.045] subscores were higher in schizophrenia patients with than in those without a right well-developed MSG in the first-episode subgroup, but not in the chronic subgroup.

The number of left short gyri positively correlated with the score for BACS motor function in the chronic subgroup (rho = 0.582, *p* < 0.001), but not in the first-episode subgroup (rho = 0.343, *p* = 0.178). A well-developed left AG was associated with better BACS executive [*F*(1, 36) = 8.30, *p* = 0.007] and motor [*F*(1, 36) = 6.70, *p* = 0.014] functions in the chronic subgroup only.

Other clinical variables (e.g., medication and illness duration) in the schizophrenia group did not correlate with anatomical variations in the insular cortex. In the ARMS group, no correlations were observed between the features of the insular gross anatomy and clinical characteristics.

## Discussion

To the best of our knowledge, this is the first MRI study to show that the number of insular gyri was higher in the ARMS and schizophrenia groups than in the control group, which indicates that they share an altered neurodevelopmental process associated with fetal gyral formation as a vulnerability factor for psychosis. The present results also revealed that well-developed insular gyri in the schizophrenia group were associated with symptom severity in the first-episode subgroup, but with preserved cognitive functions in the chronic subgroup. Therefore, the gross anatomy of the insular cortex appears to reflect state abnormalities associated with early neurodevelopment, while its contribution to the clinical characteristics of schizophrenia may differ according to the illness stages.

The present results are consistent with the findings of previous study (7), which showed that the number of insular gyri was higher in schizophrenia patients than in healthy controls regardless of the illness stages (i.e., both first-episode and chronic stages). We also revealed that the features of the insular gross anatomy were similar between schizophrenia and ARMS. The results obtained herein are also consistent with previous post-mortem (2–4, 6) and MRI (3, 5, 34) findings from non-clinical populations showing that the ASG, PSG, and ALG consistently developed in human brains, whereas the degree of development of the AG, MSG, and PLG varied more (2–6). Although the mechanisms regulating fetal insular gyration remain unclear, insular gyral patterns are formed between 17 and 35 weeks of gestation (10, 11), potentially along with the synaptogenesis and development of local neuronal connectivity (35), but remain rather stable after birth (36). In combination with previous findings showing shared gross brain characteristics between ARMS and schizophrenia [e.g., surface morphology of the orbitofrontal cortex (17, 37), Heschl’s gyrus duplication pattern (16), and diverse cortical hyper-gyrification (15)], the present insular results support brain gyrification patterns representing a static neurodevelopmental pathology associated with general vulnerability to psychosis (19, 38). On the other hand, as suggested in previous studies (15, 37), ARMS individuals who later develop psychosis may have prominent brain anomalies during early neurodevelopment (39). The present study did not examine the relationship between the gross insular anatomy and a later onset of psychosis due to the small number of ARMS individuals who developed psychosis during the follow-up (*N* = 5). Nevertheless, our result showing an over-developed left posterior insula, which develops later than the right (11) or anterior (10) subregion during fetal gyral formation, in schizophrenia but not in ARMS suggests a relationship between prolonged early neurodevelopmental insults and the later development of psychosis.

Despite the small sample size in the first-episode subgroup (*N* = 17), the results obtained herein were consistent with our previous findings from an independent and larger cohort (7) showing that well-developed insular gyri in the anterior subdivision contributed to positive psychotic symptoms specifically during the early stages of schizophrenia. Although the functional significance of the insular gross morphology remains largely unknown, brain hyper-gyrification in schizophrenia is generally considered to be associated with neural dysconnectivity due to abnormal neurodevelopmental processes (19, 38). Therefore, as discussed elsewhere (7), the present results support previous findings on first-episode schizophrenia showing relationships between the severity of positive symptoms and hyper-gyrification (20), functional dysconnectivity (40), and cortical dysfunction (41) in the anterior insular subdivision. Similar dysconnectivity or decreased white matter integrity involving the anterior insula (42, 43) and its relationship with symptom severity (44) were previously reported in ARMS individuals, particularly those who later developed psychosis (44). Collectively, these findings and the present results support the insular model of psychosis, in which a dysfunction in the anterior insular salience network interacts with central executive and default mode networks (45) and plays a critical role in psychosis by causing self-monitoring errors, heightened uncertainty, and deficits in information processing (46). The insular gross anatomy was associated with clinical symptoms only in the first-episode schizophrenia subgroup in the present study, which may be partly explained by the subthreshold and/or non-specific psychopathology of the ARMS group (47) and various factors affecting clinical symptoms at later stages of schizophrenia (e.g., medication effects, illness chronicity, and environmental factors) (48). It may be also possible that more prominent MSG development in schizophrenia than in ARMS groups (Figure 2) partly accounts for their different symptomatology.

One of the primary purposes of the present study was to investigate the relationships between the features of the insular gross anatomy and cognitive functions in the ARMS group and first-episode and chronic schizophrenia subgroups. The insular cortex, a component of the limbic integration cortex (1), has sulcally defined and functionally different subdivisions (2); the anterior subdivision is involved in a range of cognitive functions including emotional, language-related, executive, and motor control functions, whereas the posterior subdivision includes somatosensory areas (1, 49). Given the high proportion of ARMS individuals without psychosis onset in this study (*N* = 52/57), our results of shared gross insular abnormalities in the anterior subdivision between first-episode schizophrenia and ARMS groups may partly support the notion that cognitive deficits, especially in social function (50) and verbal fluency (22), exist in ARMS group irrespective of psychosis onset as a trait vulnerability marker. However, the insular gross anatomy was not associated with cognitive function in these subjects, suggesting that the factors associated with fetal insular development alone could not explain their cognitive deficits. On the other hand, the present results revealed that a well-developed gyral pattern in the anterior cognitive subdivision was associated with ‘preserved’ function for executive and motor subdomains specifically in the chronic stage of schizophrenia. While the insular gross anatomy is considered to be a stable neurodevelopmental marker of psychosis, as discussed above, the insular cortex is a brain region that exhibits active gray matter reductions (i.e., 3–5%/year) during the early stages of psychosis (8, 9). Despite the cross-sectional design of the present study, direct comparisons of insular developmental patterns between the ARMS and schizophrenia groups (Figure 2) suggested that an underdeveloped AG and MSG at a high-risk status may change to an absent pattern due to the above-described progressive gray matter reduction. Since active gray matter reduction in the early phase of schizophrenia may be associated with cognitive decline (51), it is possible that patients with less severe gray matter changes, who have well-developed insular gyri even at later illness stages, are characterized by preserved cognitive functions in the chronic stage. This hypothesis needs to be examined in future longitudinal studies, ideally in combination with functional/connectivity neuroimaging.

The present study has several potential limitations that need to be addressed. First, the present study systematically assessed cognitive functions in the ARMS and schizophrenia groups, but not in the control group. Correlations were observed between variations in the gross insular anatomy and executive and motor functions in the schizophrenia group; however, the insular cortex (particularly the anterior subdivision) plays a role in a number of other cognitive domains, such as emotional, auditory processing, and language-related functions (49). Since a large inter-individual anatomical variation in insular gyri was also observed in the control group, its general relationship with cognitive and other brain functions warrants further study. Second, the ARMS group in this study was significantly younger than the other groups. However, it is unlikely that this age difference significantly affected the gross gyral organization, which is a stable brain characteristic. Further, the present results showing group differences in the insular gross anatomy remained unchanged after adjustments for age as a covariate. The present study was also limited by the small sample size of ARMS individuals who subsequently developed psychosis (*N* = 5). Therefore, the relationships between the features of the gross insular anatomy and the later onset of psychosis warrants further study in larger high-risk cohorts. Third, the duration of untreated psychosis (DUP), a clinical factor that significantly influences brain morphology/function and clinical course of schizophrenia (52), was not systematically assessed in this study. While the gross anatomical features of the insular cortex were not influenced by illness duration of the patients in this study, potential influence of DUP on our results should be tested in future studies. Finally, since alterations in gross brain characteristics (e.g., gyrification pattern) have also been reported in other major psychiatric diseases, such as bipolar and autism spectrum disorders (19), further studies will be required to examine the disease specificity of the gross insular findings identified in the present study.

In conclusion, the present MRI study investigated gross anatomical variations in the insular cortex and the results obtained support similar brain characteristics between schizophrenia patients and clinical high-risk individuals, which may be attributed to common vulnerability associated with early neurodevelopmental abnormalities. However, the contribution of the insular gross anatomy to the clinical characteristics of schizophrenia (i.e., symptom severity and cognitive functions) appeared to differ according to the illness stages. To clarify the complex insular pathology of psychosis, further studies are needed on the functional significance and potential factors (e.g., active gray matter changes) associated with this gross anatomical variation.

## Data availability statement

The raw data supporting the conclusions of this article will be made available by the authors, without undue reservation.

## Ethics statement

The studies involving human participants were reviewed and approved by Committee of Medical Ethics of the University of Toyama. Written informed consent to participate in this study was provided by the participants’ legal guardian/next of kin.

## Author contributions

MS, YH, NT, and TT conceived the concept and methodology of the present study. TT conducted the statistical analyses and wrote the manuscript. DS, YT, YH, HK, YM, SN, and YA recruited subjects and were involved in clinical and diagnostic assessments. TT and DS analyzed the MRI data. KN provided the technical support for MRI scanning and data processing. AF and YY managed the MRI and clinical data. MS and YT contributed to the writing and editing of the manuscript. All authors contributed to and approved the final manuscript.

## Funding

This work was supported by JSPS KAKENHI Grant Numbers JP18K07550 to TT, JP18K15509 to DS, JP23K07031 to NT and JP20H03598 to MS, and by Health and Labour Sciences Research Grants for Comprehensive Research on Persons with Disabilities from the Japan Agency for Medical Research and Development (AMED) Grant Numbers JP19dk0307029 to MS and JP22dk0307103h0002 to TT. These funding sources had no role in the study design; in the collection, analysis, and interpretation of data; in the writing of the manuscript; or in the decision to submit the manuscript for publication.

## Conflict of interest

The authors declare that the research was conducted in the absence of any commercial or financial relationships that could be construed as a potential conflict of interest.

## Publisher’s note

All claims expressed in this article are solely those of the authors and do not necessarily represent those of their affiliated organizations, or those of the publisher, the editors and the reviewers. Any product that may be evaluated in this article, or claim that may be made by its manufacturer, is not guaranteed or endorsed by the publisher.
